# Correction: Lectin recognizing thermoresponsive double hydrophilic glycopolymer micelles by RAFT polymerization

**DOI:** 10.1039/d6ra90057g

**Published:** 2026-05-26

**Authors:** Kan Sun, S. W. Annie Bligh, Hua-li Nie, Jing Quan, Li-min Zhu

**Affiliations:** a College of Chemistry, Chemical Engineering and Biotechnology, Donghua University Shanghai, 201620 P.R. China lzhu@dhu.edu.cn +86 21 67792748; b Department of Life Sciences, Faculty of Science and Technology, University of Westminster 115 New Cavendish Street London W1W 6UW UK

## Abstract

Correction for ‘Lectin recognizing thermoresponsive double hydrophilic glycopolymer micelles by RAFT polymerization’ by Kan Sun *et al.*, *RSC Adv.*, 2014, **4**, 34912–34921, https://doi.org/10.1039/C4RA04874A.

The authors regret that the Fig. 1(c) NMR trace showed various unexpected features. This was caused by the data processing software Origin and graphing software Adobe Illustrator and went undetected during verification. Furthermore, we regret that the incorrect chemical structure of OVAG was shown in Fig. 1(c). The correct NMR spectrum and chemical structure are shown below.

**Fig. 1:**
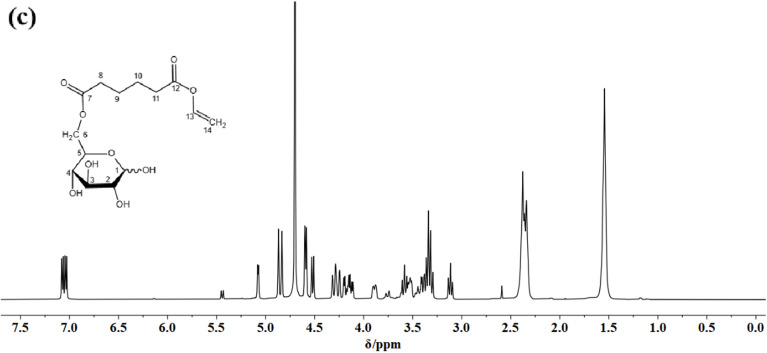
(c) ^1^H NMR spectrum of OVAG in D_2_O.

An independent expert has reviewed the raw NMR data and the original and new images and has concluded that they are consistent with the discussion and conclusions presented.

The Royal Society of Chemistry apologises for these errors and any consequent inconvenience to authors and readers.

